# Réaction à Moujahid et al.: Les kystes hydatiques du foie rompus dans les voies biliaires: à propos de 120 cas

**Published:** 2012-02-28

**Authors:** Mahdi Bouassida, Mohamed Mongi Mighri, Mechaal Benali, Fathi Chebbi, Hassen Touinsi, Sadok Sassi

**Affiliations:** 1 Service de chirurgie générale, Hôpital Mohamed Tahar Maamouri Nabeul, Tunisia; 2 Service d’anesthésie-Réanimation, Hôpital Mohamed Tahar Maamouri Nabeul, Tunisia

**Keywords:** Kyste hydatique, foie, complications biliaires, cholangite sclérosante

## Réaction

Nous avons lu avec intérêt l’article de Moujahid et al. portant sur les kystes hydatiques du foie rompus dans les voies biliaires: à propos de 120 cas [1]. Nous avons trouvé qu’il s’agissait de l’une des plus grandes séries rapportées dans la littérature, concernant cette complication redoutable du kyste hydatique du foie. Néanmoins, nous avons jugé que quelques remarques sont à faire. En effet, les auteurs n’ont pas fait allusion à la classique distinction entre : fistulettes, dont la taille est inférieure à 5 mm, et larges fistules kysto-bilaires dont la taille est supérieure à 5 mm. En effet, cette distinction demeure d’actualité vu que le pronostic et la prise en charge de ces deux entités sont différents [2]. Ce sont les larges fistules kysto-biliaires qui se compliquent en général de migration de matériel hydatique dans les voies biliaires, ce qui est responsable d’épisodes d’angiocholites aigues parfois graves. Les fistulettes sont par contre responsables de passage de bile dans la cavité kystique et à la limite d’infection du kyste hydatique. Sur le plan chirurgical, les fistulettes sont sujettes à une suture simple associée à un drainage de la cavité kystique, alors que les larges fistules posent le plus de problèmes de prise en charge.

Un deuxième point qui mériterait d’être discuté : la stérilisation du kyste, en présence de fistules kysto-biliaires. En effet, plusieurs études ont montré le risque de cholangite sclérosante secondaire due aux scolicides [3,4]. Ce risque est certes moindre avec le sérum salé hypertonique, en comparaison avec les autres agents scolicides, mais demeure réel, d’autant plus qu’il a été vérifié sur les modèles animaux [5–7]. Malgré l’absence de recommandations précises à ce sujet, nous déconseillons l’injection de scolicide dans la cavité kystique, en cas de suspicion de fistules kysto-biliaires.

Sur le plan thérapeutique, les auteurs ont évoqué la suture simple, associée au drainage, comme étant source de fuites biliaires. En effet, ceci dépond de l’état du périkyste : si ce dernier est souple, la suture est en général dénuée de risque de lâchage, alors que si le périkyste est épais, la suture serait dérisoire, sinon utopique. Pour pallier à ce problème, nous conseillons, le cas échéant, le recours à une périkystectomie périfistulaire qui améliorerait les résultats de cette méthode [8]. Enfin, mis à part la suture simple associée ou non au drainage et la déconnexion kysto-biliaire, une troisième méthode pour le traitement des larges fistules kysto-biliaires mériterait d’être évoquée: le drainage interne trans-fistulo-oddien (DITFO) qui a été prôné par plusieurs auteurs avec des résultats satisfaisants [8].
